# Effects of reallocating time in different activity intensities on health and fitness: a cross sectional study

**DOI:** 10.1186/s12966-015-0249-6

**Published:** 2015-06-24

**Authors:** Daniel Aggio, Lee Smith, Mark Hamer

**Affiliations:** Health Behaviour Research Centre, University College London, 1-19 Torrington Place, WC1E 6BT London, UK; Department of Epidemiology and Public Health, Physical Activity Research Group, University College London, 1-19 Torrington Place, WC1E 6BT London, UK; National Centre Sport & Exercise Medicine, Loughborough University, Loughborough, UK

**Keywords:** Sedentary behaviour, Physical activity, Accelerometry, Isotemporal substitution, Fitness

## Abstract

**Background:**

The effects of replacing time in specific activity categories for other categories (e.g. replacing sedentary time with light activity) on health and fitness are not well known. This study used isotemporal substitution to investigate the effects of substituting activity categories in an equal time exchange fashion on health and fitness in young people.

**Methods:**

Participants were drawn from schools in Camden, London (*n* = 353, mean age 9.3 ± 2.3 years). Time sedentary, in light and in moderate-to-vigorous activity (MVPA) was measured via accelerometry. The effects of substituting time in activity categories (sedentary, light and MVPA) with equivalent time in another category on health and fitness were examined using isotemporal substitution.

**Results:**

In single and partition models, MVPA was favourably associated with body fat %, horizontal jump distance and flexibility. Time sedentary and in light activity were not associated with health and fitness outcomes in these models. In substitution models, replacing one hour of sedentary time with MVPA was favourably associated with body fat % (B = −4.187; 95 % confidence interval (CI), −7.233, −1.142), horizontal jump distance (B = 16.093; 95 % CI, 7.476, 24.710) and flexibility (B = 4.783; 95 % CI, 1.910, 7.656). Replacing time in light activity with MVPA induced similar benefits but there were null effects for replacing sedentary with light intensity.

**Conclusion:**

Substituting time sedentary and in light activity with MVPA was associated with favourable health and fitness. Time in sedentary behaviour may only be detrimental to health and fitness when it replaces time in MVPA in young people.

**Electronic supplementary material:**

The online version of this article (doi:10.1186/s12966-015-0249-6) contains supplementary material, which is available to authorized users.

## Introduction

Regular participation in physical activity aids in the prevention against risk factors for non-communicable disease in young people [1], and promotes cardiorespiratory fitness (CRF) [2] and muscular strength [3]. Some research in young people suggests that only vigorous intensity activity can elicit improvements to cardiometabolic health [4], but there is also some evidence showing favourable associations with light intensity activity [5]. One possible explanation for these inconsistent findings may be due to the varying methodologies used in accelerometry research. Different motion sensors, wear positions, data processing methods and/ or differing cut points to categorise intensity of activity are issues that make direct comparison challenging in this field [6]. Sedentary behaviour research has also highlighted associations with several cardiometabolic health risk factors independent of physical activity levels in young people [7], although evidence remains inconsistent [8]. Moreover, evidence suggests that high amounts of sedentary behaviour in childhood are related to lower CRF in adolescence [9].

Fitness levels are indicative of habitual physical activity in young people [2], but there is little evidence for how different intensity categories are independently associated with components of fitness. Some evidence suggests that only vigorous intensity activity can elicit improvements in CRF [4, 10] but associations with other components of fitness such as strength and power remain unreported despite being strongly associated with child health [11–13]. Flexibility tracks from childhood into adulthood [14] and is important for reducing the risk of chronic musculoskeletal problems in later life [15]. Although it is plausible to assume that specific types of activity (e.g. gymnastics) and higher overall activity levels elicit more favourable levels of flexibility, little is known about what intensity of activity can induce such benefits. Further work is required to elucidate how different intensities of activity determine these components of fitness.

Regular participation in physical activity and minimising sedentary behaviours promotes health and fitness in young people [16, 17] but it is unclear how the reallocation of time from one activity intensity to another might affect health and fitness outcomes. For example, would similar health benefits be achieved if sedentary time was replaced with an equal amount of time in light intensity activity as opposed to replacing it with time in moderate-to-vigorous physical activity (MVPA)? If so, increasing light activity may be a more practical and achievable intervention target to induce change at the population level rather than seeking to increase levels of MVPA which may be more challenging to modify [18].

Using isotemporal substitution, a technique that can simultaneously model the effect of displacing one activity for an equivalent amount of time in another activity, recent research has started to explain the effects of time reallocation of activity intensities on health in adults [19–21]. Findings showed that replacing sedentary time with equivalent amounts of MVPA yielded associated health benefits [19]. Other studies in adults have also demonstrated benefits of replacing sedentary time with light activity [20]. This technique has yet to be applied to youth populations and the effects of time reallocation on fitness levels remain unreported. The aim of this study was to use the isotemporal substitution technique to investigate the displacement effect when time in one activity category is replaced with equal time in another category on a range of health and fitness outcomes in young people.

## Methods

Participants (*n* = 450) were aged between 5 and 15 years and recruited from nine schools (7 primary and 2 secondary) from the London borough of Camden, UK, as part of the Camden Active Spaces project [22]. Head teachers provided written consent for all young people to take part. Parental consent was obtained using an opt-out approach. Participants in secondary schools were also asked to provide explicit written assent. Ethical approval was granted by the University College London Research Ethics Committee (Reference number: 4400/002).

### Physical activity and sedentary time assessment

Physical activity and sedentary time were measured objectively with tri-axial accelerometers (Actigraph wGT3X-BT), which are deemed valid and reliable for measuring these behaviours in young people [23]. Participants were instructed to wear these devices continuously on the right hip for seven consecutive days during waking hours apart from when engaging in water-based activities. Data were downloaded using Actilife (version 6) software. Age-specific cut points were used to determine time spent sedentary, in light and in MVPA [8]. Less than 100 accelerometer counts per minute (cpm) was classified as sedentary and minutes with more than 3000 cpm as time in MVPA [8]. Sixty minute bouts or more of continuous zero counts were excluded from the data and considered as non-wear time [24]. After exclusion of the first day (a partial day of wear time), data were then exported into excel. Daily averages for time (hours) spent sedentary, in light and in MVPA were calculated from valid days. Valid days required 500 min of wear time between 07:00 and 00:00. Participants with no valid days were excluded from analyses. These inclusion criteria have previously been used in studies of this kind [8].

### Health and fitness outcomes

The following fitness tests were chosen as they have been extensively used in previous cohort studies of young people [25–27], which were used to develop protocols for the present study. Body fat (%) was measured using a Tanita SC-330 Body Composition Analyser (Tanita Inc, Illinois, USA). Hand grip strength of the dominant arm was determined using a hand-held Dynamometer (JAMAR®, Hydraulic Hand Dynamometer). Participants held the dynamometer in a standing positon and were instructed to squeeze the grip as hard as possible. Leg power was measured via a horizontal jump test. From a standing position participants were instructed to bend their knees and use their arms to jump as far forward as possible onto a landing mat. Flexibility was determined using a sit-and-reach test using a standard sit-and-reach box. Participants removed shoes and were asked to keep their knees fully extended whilst slowly leaning as far forward as possible and sliding the ruler along the surface of the box with their fingertips. Peak expiratory flow was assessed using a peak flow meter. Participants completed each test three times and the best score was recorded. Trained research assistants supervised all measurements, which were completed on school premises.

### Covariates

Participants reported their age, sex and ethnicity. School postcodes were used to derive school deprivation. Geoconvert [28] was used to translate postcodes into an Indices of Deprivation 2007 lower layer super output areas (LSOA) Score. Schools were then ranked in order of deprivation. Stature was measured to the nearest 0.1 cm using a Leicester Height Measure with participants in the Frankfort plane.

### Analyses

Linear regression models were used to examine associations between time (hours /day) in sedentary, light and MVPA with health and fitness outcomes. Time in different activity intensities was converted to hours/d, so that coefficients were more easily interpreted relative to the current guidelines (60 min per day of MVPA for young people aged 5 to 17). Models were adjusted for pre-specified covariates hypothesised to be independently associated with both exposure and outcome variables, including age, sex, ethnicity, deprivation and height. Three different linear regression models were used, which are reported elsewhere [19]. In brief, the first models are single-factor examining the association of each intensity category (sedentary and light activity and MVPA) with health and fitness outcomes without mutual adjustment for other categories of activity. The second are partition models examining the association of each intensity category whilst controlling for the other categories of activity but not total device wear time. The third are isotemporal substitution models representing the estimated effects of substituting one intensity category for the category dropped whilst controlling for total wear time. For example, in the isotemporal model examining the effect of substituting sedentary time with time in light activity or in MVPA, the model dropped sedentary time but included time in light activity, MVPA, total wear time and other covariates. These models assume linear relationships between dependent and independent variables which were determined prior to running these analyses. Analyses were conducted using SPSS version 22.

## Results

From the initial sample (*n* = 450), final analyses contained 353 participants (male, 47.9 %; mean age 9.3 ± 2.3 years). The sample was reduced due to missing health/fitness data (*n* = 33), covariates (*n* = 43) and accelerometry data (*n* = 22 device not returned; *n* = 33 insufficient wear time). In the analytic sample, all participants provided at least one valid day, with over half of those (55.2 %) providing at least five valid days and just 7.4 % providing only one valid day. Participants excluded from analyses were younger (8.3 years vs. 9.3 years, *p* < 0.05), shorter in stature (133.1 cm vs. 139.1 cm, *p* < 0.05), had lower levels of body fat (20.1 % vs. 22.6 %, *p* < 0.05) and were more likely to be female (62.2 % vs. 52.1 %, *p* < 0.05) when compared to the analytic sample. Sample characteristics are presented in Table 1, including health and fitness outcomes and objectively measured activity levels. We observed modest correlations between each of the different activity categories (data shown in Additional file 1: Table S1).

**Table 1:** Sample characteristics (*n* = 353)

	Mean ± SD
Ethnicity (% White British)	35.7 %
Body fat (%)	22.6 ± 8.0
Horizontal jump (cm)	120.0 ± 24.3
Hand grip strength (kg)	16.3 ± 7.3
Sit and reach (cm)	25.8 ± 7.3
Peak flow (l)	217.2 ± 75.3
Sedentary (mins/day)	364.5 ± 88.3
Light (mins/day)	357.0 ± 72.0
MVPA (mins/day)	27.8 ± 16.2
Total accelerometer wear time (mins/day)	749.2 ± 94.6

Tables 2, 3, 4, 5 and 6 present single, partition and isotemporal substitution models for the associations between specific activity categories and health/fitness outcomes. In single and partition models, only MVPA was favourably associated with any health/fitness outcomes, including body fat %, horizontal jump distance and flexibility. In isotemporal substitution models, replacing sedentary activity with MVPA was associated with favourable effects on body fat %, horizontal jump distance and flexibility. Replacing light activity with MVPA was also associated with favourable effects on these outcomes. Replacing sedentary activity with light activity was not associated with any positive effect on health/fitness outcomes. For all three models, there was no association between any activity category with hand grip strength and peak flow.

**Table 2:** Single, partition, and isotemporal substitution models examining the relation between changes in time spent (hours /day) in sedentary, light and moderate-to-vigorous intensity activity and body fat % (*n* = 353)

Models	Sedentary, *B* (95 % CI)	Light activity, *B* (95 % CI)	MVPA, *B* (95 % CI)
Single	−0.422 (−1.129, 0.286)	0.076 (−0.613, 0.765)	−4.183 (−7.196, −1.171)
Partition	−0.619 (−1.337, 0.099)	0.155 (−0.538, 0.847)	−4.807 (−7.898, −1.716)
Isotemporal substitution			
Replace sedentary	Dropped	0.774 (−0.167, 1.714)	−4.187 (−7.233, −1.142)
Replace light activity	−0.774 (−1.714, 0.167)	Dropped	−4.961 (−8.212, −1.710)
Replace MVPA	4.187 (1.142, 7.233)	4.961 (1.710, 8.212)	Dropped

Regression coefficients (95 % CI) were adjusted for age, sex, ethnicity, height and school deprivation

**Table 3:** Single, partition, and isotemporal substitution models examining the relation between changes in time spent (hours p/day) in sedentary, light and moderate-to-vigorous intensity activity and hand grip strength (*n* = 353)

Models	Sedentary, *B* (95 % CI)	Light activity, *B* (95 % CI)	MVPA, *B* (95 % CI)
Single	−0.338 (−0.717, 0.041)	0.258 (−0.111, 0.627)	0.603 (−1.031, 2.236)
Partition	−0.298 (−0.687, 0.091)	0.211 (−0.164, 0.586)	0.212 (−1.462, −1.887)
Isotemporal substitution			
Replace sedentary	Dropped	0.509 (0.000, 1.019)	0.511 (−1.139, 2.161)
Replace light activity	−0.509 (−1.019, 0.000)	Dropped	0.002 (−1.760, 1.763)
Replace MVPA	−0.511 (−2.161, 1.139)	−0.002 (−1.763, 1.760)	Dropped

Regression coefficients (95 % CI) were adjusted for age, sex, ethnicity, height and school deprivation

**Table 4:** Single, partition, and isotemporal substitution models examining the relation between changes in time spent (hours p/day) in sedentary, light and moderate-to-vigorous intensity activity and horizontal jump distance (*n* = 353)

Models	Sedentary, *B* (95 % CI)	Light activity, *B* (95 % CI)	MVPA, *B* (95 % CI)
Single	−0.677 (−2.693, 1.338)	1.078 (−0.880, 3.036)	16.477 (7.989, 24.965)
Partition	0.141 (−1.891, 2.173)	0.550 (−1.410, 2.510)	16.234 (7.488, 24.981)
Isotemporal substitution			
Replace sedentary	Dropped	0.409 (−2.252, 3.070)	16.093 (7.476, 24.710)
Replace light activity	−0.409 (−3.070, 2.252)	Dropped	15.684 (6.484, 24.885)
Replace MVPA	−16.093 (−24.710, −7.476)	−15.684 (−24.885, −6.484)	Dropped

Regression coefficients (95 % CI) were adjusted for age, sex, ethnicity, height and school deprivation

**Table 5:** Single, partition, and isotemporal substitution models examining the relation between changes in time spent (hours p/day) in sedentary, light and moderate-to-vigorous intensity activity and peak expiratory flow (*n* = 353)

Models	Sedentary, *B* (95 % CI)	Light activity, *B* (95 % CI)	MVPA, *B* (95 % CI)
Single	−1.942 (−7.473, 3.589)	−1.868 (−7.248, 3.511)	3.572 (−20.210, 27.354)
Partition	−2.112 (−7.799, 3.575)	−2.261 (−7.746, 3.224)	3.277 (−21.202, 27.757)
Isotemporal substitution			
Replace sedentary	Dropped	−0.149 (−7.569, 7.298)	5.389 (−18.728, 29.506)
Replace light activity	0.149 (−7.298, 7.596)	Dropped	5.538 (−20.210, 31.287)
Replace MVPA	−5.389 (−29.506, 18.728)	−5.538 (−31.287, 20.210)	Dropped

Regression coefficients (95 % CI) were adjusted for age, sex, ethnicity, height and school deprivation

**Table 6:** Single, partition, and isotemporal substitution models examining the relation between changes in time spent (hours p/day) in sedentary, light and moderate-to-vigorous intensity activity and flexibility (*n* = 353)

Models	Sedentary, *B* (95 % CI)	Light activity, *B* (95 % CI)	MVPA, *B* (95 % CI)
Single	−0.037 (−0.706, 0.633)	0.306 (−0.344, 0.957)	4.926 (2.095, 7.757)
Partition	0.215 (−0.462, 0.893)	0.167 (−0.487, 0.820)	4.998 (2.082, 7.914)
Isotemporal substitution			
Replace sedentary	Dropped	−0.048 (−0.935, 0.839)	4.783 (1.910, 7.656)
Replace light activity	0.048 (−0.839, 0.935)	Dropped	4.831 (1.764, 7.899)
Replace MVPA	−4.783 (−7.656, −1.910)	−4.831 (−7.899, −1.764)	Dropped

Regression coefficients (95 % CI) were adjusted for age, sex, ethnicity, height and school deprivation

## Discussion

This study aimed to investigate how the displacement of activity intensities is associated with health and fitness outcomes in young people using the isotemporal substitution technique, which until now has yet to be used in these populations. Our results demonstrate that replacing sedentary time or light intensity activity with an equivalent amount of MVPA was associated with more favourable health and fitness outcomes in young people. Our findings also showed no beneficial effect of replacing sedentary time with light intensity activity, which is in concert with some, [19] but not all previous work in adults using the isotemporal substitution technique [20]. The substitution model adjusts for time spent in other activity intensities whilst also controlling for total wear time, thus associations between activity intensities and health and fitness are independent of each other and of total wear time. Similar research in young people has also suggested that only MVPA or vigorous activity can elicit benefits to health and CRF [4, 10]. Our study supports these findings, albeit with other components of fitness, such as flexibility and leg power. Taken together with our findings, these studies are in contrast with other research that demonstrates some beneficial effect of light intensity activity with cardiometabolic health in young people [5]. A plausible explanation for these discrepancies may be the varying cut off points used to define light activity in these studies. The benefits of light physical activity may be more pronounced in less fit and inactive populations.

Some research in children and adolescents has shown sedentary behaviour to be negatively associated with some health and fitness outcomes independently of physical activity levels, [7, 29] but our data did not support this hypothesis. A possible explanation for the discrepancy in findings may be the methodological differences in the assessment of physical activity/sedentary time. We objectively measured physical activity using accelerometry, whereas some other studies have used self- or parent-reported data. There were also differences in the health and fitness outcomes measured that may also explain the discrepancies between studies.

In single and partition models, sedentary time was not associated with any health and fitness outcomes. Only in the substitution model, when MVPA was replaced by sedentary time were negative effects visible. These findings are consistent with the notion that associations between sedentary behaviour and health are largely driven because they displace time spent being physically active [19, 30]. The markedly inconsistent data in relation to sedentary time and health outcomes in children might be explained by different sitting patterns compared with adults. For example, adults may record more bouts of prolonged sitting compared to children who may conceivably record a higher frequency of short sitting periods supplemented with movement.

Although MVPA was associated with body fat %, flexibility and leg power, there was no association with some components of fitness, namely handgrip strength and peak expiratory flow. Even when MVPA displaced sedentary or light activity in the substitution models there were no significant associations, which is somewhat surprising given the strong association reported between these outcomes and health [11, 31]. This highlights the importance of promoting muscle strengthening activities as well as attaining 60 min of MVPA in physical activity guidelines. The lack of associations with these components of fitness may be partly explained by the inability of accelerometers to capture some activities that may be beneficial for these types of fitness (eg swimming that develops lung capacity). Another methodological limitation of accelerometers is that they may be less accurate at distinguishing between sedentary and light activities than they are for categorising MVPA levels. Methods that can detect postural allocation such as the ActivPal may be more reliable for determining between sitting and light activities, which may explain why replacing sedentary time with light activity had no beneficial effect in our study. Other studies have sub-categorised light intensity activity into low-light and high-light intensity activity, with time in the higher end showing additional benefits to health than time in the lower end of light activity [5]. Further categorisation of light activity may therefore have yielded some potential benefit in the present study. Furthermore, accelerometers cannot capture activity during non-wear time, therefore activities during these times are not accounted for. Criteria for classifying accelerometer non-wear time vary in the literature [24, 32]. For the present study 60 min of continuous zero counts were used to define non-wear. It seems reasonable to assume that 5–15 year olds are unlikely to remain sufficiently stationary to register no movement on an accelerometer for more than 60 min when awake, and this criterion has been applied in previous studies [8, 24]. In addition, the isotemporal substitution technique is a statistical model that cannot reflect true time reallocation, and as with all cross-sectional research we cannot determine causality. We cannot rule out the possibility of reverse causation, in that children who are fitter may in turn be more active. Nevertheless, these data are the first to show the beneficial effect of displacing time spent sedentary or in light activity with MVPA on body fat, flexibility and leg power in young people. Further research should continue to explore the effects of specific activity categories on health and be repeated with a longitudinal design and measures more appropriate for distinguishing between light and sedentary activities.

In conclusion, the isotemporal substitution technique demonstrated that substituting sedentary time with MVPA only, and not light activity, was associated with more favourable health and fitness outcomes in young people. Time in sedentary behaviour may only be detrimental to health and fitness when it replaces time in MVPA.

## Additional file

Additional file 1: Table S1. Correlation matrix for accelerometry variables.

## Abbreviations

CRF: Cardiorespiratory fitness
MVPA: Moderate-to-vigorous physical activity
CPM: Counts per minute
LSOA: Lower layer super output areas
