# Supplementary material for: Effects of reallocating time in different activity intensities on health and fitness: a cross sectional study
Source: Int J Behav Nutr Phys Act. 2015 Jun 24;12:83. doi: 10.1186/s12966-015-0249-6 (PMC4482052; doi:10.1186/s12966-015-0249-6)
Supplement: Additional file 1: Table S1. — Correlation matrix for accelerometry variables. [file 12966_2015_249_MOESM1_ESM.docx]

**Supplementary table 1.** Correlation matrix for accelerometry variables

|  | **Wear time** | **Sedentary** | **Light** | **Moderate-to-vigorous** |
| --- | --- | --- | --- | --- |
| **Wear time** | 1 |  |  |  |
| **Sedentary** | 0.65** | 1 |  |  |
| **Light** | 0.50** | -0.31** | 1 |  |
| **Moderate-to-vigorous** | 0.04 | -0.27** | 0.17* | 1 |

*p<0.01;**p<0.001
